# Explainable AI for early developmental disability detection: a neuro-fuzzy approach

**DOI:** 10.3389/fpubh.2026.1691449

**Published:** 2026-05-13

**Authors:** Adel Saber Alanazi, Sohil Alqazlan, Rayan Alanazi, Houcine Benlaria

**Affiliations:** 1 College of Education, Jouf University, Sakakah, Saudi Arabia; 2 College of Education, Imam Mohammad Ibn Saud Islamic University (IMSIU), Riyadh, Saudi Arabia; 3 College of Computer and Information Sciences, Jouf University, Sakakah, Saudi Arabia; 4 College of Business, Jouf University, Sakakah, Saudi Arabia

**Keywords:** Adaptive Neuro-Fuzzy Inference System (ANFIS), autism spectrum disorder, developmental disabilities, early intervention, explainable AI, fuzzy logic, interpretable machine learning, pediatrics diagnosis

## Abstract

**Background:**

Developmental disabilities affect approximately 1 in 6 children aged 3–17 years. The diagnostic process typically spans 2–3 years and disproportionately affects underserved populations. Traditional machine learning approaches for screening have demonstrated promising accuracy but often lack the transparency required for clinical acceptance.

**Methods:**

We developed and validated an Adaptive Neuro-Fuzzy Inference System (ANFIS) using a longitudinal observational dataset of 5,000 children aged 1–6 years from 12 early childhood centers (4,311 typically developing; 689 with developmental disabilities, prevalence 13.8%). The ANFIS model implemented Gaussian membership functions and Sugeno fuzzy inference, incorporating age-adjusted ratios and cross-domain interaction features. Model performance was evaluated using standard binary classification metrics and five-fold stratified cross-validation.

**Results:**

All four assessment domains differed significantly between diagnostic groups (*p* < 0.001): Cognitive Scores (60.86 ± 14.51 vs. 51.58 ± 17.09), Behavioral Scores (55.82 ± 9.38 vs. 49.64 ± 11.21), Motor Skills (51.08 ± 7.79 vs. 45.08 ± 8.00), and Social Interaction (51.20 ± 11.66 vs. 42.13 ± 11.19). Family history was present in 85.6% of diagnosed children vs. 45.0% of typically developing children (*p* < 0.001). The ANFIS model achieved 96.0% accuracy, 87.5% sensitivity, 97.6% specificity, and AUC = 0.925 on a held-out test set of 100 cases. Five-fold cross-validation yielded a mean accuracy of 89.2% ± 3.4% (95% CI: [85.8, 92.6%]). Cognitive_Social_Ratio was the most influential diagnostic indicator.

**Conclusion:**

The ANFIS approach demonstrates a clinically relevant balance between diagnostic accuracy and interpretability, positioning it as a viable clinical decision-support tool. Larger external validation studies across diverse populations are required before widespread implementation.

## 1 Introduction

Developmental disabilities—encompassing autism spectrum disorders, intellectual disabilities, language and communication delays, and specific developmental impairments—represent one of the most significant public health challenges in pediatric medicine. In the United States alone, approximately 1 in 6 children aged 3–17 years is affected, with global prevalence estimates continuing to rise as surveillance methods improve (1, 2). The consequences extend well beyond the individual: annual economic costs in the United States exceed $61 billion, and early intervention services have demonstrated cost-effectiveness ratios of up to 1:7 relative to lifetime care costs, underscoring the profound public health and economic case for timely identification (47). The neuroplasticity of the developing brain is most pronounced during the initial six years of life, indicating that the timing of diagnosis directly influences the efficacy of intervention; even brief delays can significantly reduce the opportunity for optimal developmental progress (3).

Despite this urgency, the diagnostic process for developmental disabilities remains protracted and inequitable. Wait times for comprehensive developmental evaluations frequently exceed 6–12 months in many regions, and extended diagnostic timelines disproportionately affect children from lower socioeconomic, racial, and ethnic minority backgrounds, compounding existing health disparities (4–8). The diagnostic challenge itself is considerable: developmental disabilities present heterogeneously, with overlapping symptom profiles, variable developmental trajectories, and frequent comorbidities that complicate differential diagnosis (9). Inter-rater reliability among experienced clinicians varies substantially, particularly for subtle presentations and borderline cases, and access to the specialized multidisciplinary teams required for comprehensive evaluation remains uneven across healthcare systems (10). These structural and clinical barriers collectively reinforce the case for decision-support tools capable of improving diagnostic consistency, efficiency, and accessibility.

The integration of machine learning into clinical diagnostics has emerged as a promising avenue for addressing these challenges. Recent systematic reviews have demonstrated that machine learning approaches can achieve 85–95% accuracy in developmental disability classification, consistently outperforming unaided clinical screening in controlled settings (11, 12). However, a fundamental problem of interpretability has substantially limited the clinical translation of these advances. Most high-performing machine learning models, such as deep neural networks and ensemble methods, function as opaque systems, providing predictions without providing explanatory reasoning (13). In clinical practice, where diagnostic decisions carry significant consequences for children and families, regulatory requirements, professional accountability, and the need for clinician-patient communication all demand that a diagnostic system be not only accurate but also transparently justified. Topol (14) has convincingly contended that the effective incorporation of artificial intelligence into medicine depends on maintaining, rather than replacing, the clinician’s capacity to comprehend and scrutinize diagnostic reasoning. This interpretability requirement represents the central unsolved challenge for machine learning in pediatric developmental assessments.

The Adaptive Neuro-Fuzzy Inference System (ANFIS) offers a principled response to this challenge. ANFIS is a hybrid computational architecture that combines the adaptive learning capabilities of neural networks with the rule-based, linguistically interpreted structure of fuzzy logic systems (15). Unlike conventional neural networks, ANFIS expresses its learned knowledge as a transparent set of if-then fuzzy rules, enabling clinicians to understand precisely which assessment domains and threshold values drive each diagnostic classification (16). The Gaussian membership functions at the core of the ANFIS architecture further allow the quantification and communication of diagnostic uncertainty—a property of particular relevance in developmental assessment, where borderline presentations are common and absolute diagnostic certainty is rarely achievable. Recent applications of neuro-fuzzy systems in medical diagnosis have demonstrated that this interpretability advantage can be achieved without meaningful sacrifice of classification accuracy relative to conventional machine learning approaches (17, 18).

This study validates an ANFIS-based diagnostic system using a multi-domain clinical dataset of 5,000 children aged 1–6 years from 12 early childhood centers, encompassing cognitive, behavioral, motor, and social interaction assessments alongside demographic and family history variables. The primary objectives were to (1) develop and validate an ANFIS model for developmental disability diagnosis using comprehensive, multi-domain assessment data; (2) evaluate model performance against clinical diagnoses established by multidisciplinary teams following standard diagnostic protocols; (3) identify the most influential factors in the diagnostic process through feature importance analysis and cross-domain interaction assessment; and (4) assess model interpretability through membership function analysis and representative clinical application scenarios. Additionally, this research aimed to provide calibrated confidence measures for diagnostic predictions, enabling clinicians to appropriately weight AI-assisted recommendations within their decision-making framework.

The results presented here aim to assist clinicians in the intricate process of diagnosing developmental disabilities, potentially decreasing diagnostic delays, enhancing consistency across various settings, and broadening access to evidence-based screening in resource-constrained environments. The transparent decision-making architecture of the ANFIS model may also facilitate regulatory approval pathways, serve as an educational tool for clinician training, and contribute to the broader evidence base for explainable AI in pediatric healthcare—a field in which early and accurate identification can fundamentally alter long-term developmental trajectories and quality of life.

## 2 Literature review

The application of machine learning to the diagnosis of developmental disabilities represents an emerging field at the intersection of clinical pediatrics, developmental psychology, and artificial intelligence. This literature review examines the current state of knowledge across three key domains: (1) challenges in developmental disability diagnosis, (2) machine learning approaches in pediatric assessment, and (3) interpretable AI and neuro-fuzzy systems in healthcare.

### 2.1 Challenges in developmental disability diagnosis

The diagnosis of developmental disability, particularly autism spectrum disorder (ASD), presents multifaceted challenges that complicate accurate evaluation. A significant problem is the limitations of existing diagnostic tools, which often struggle to distinguish between ASD and other neurodevelopmental disorders owing to overlapping symptoms and variable presentations (19, 20). Tools such as the DSM-5 have evolved, but the diagnostic criteria may not capture the entire spectrum of ASD presentations, particularly in different populations, such as young women and girls, who can exhibit subtler signs (21–23).

The variability in development observed in children requires a nuanced approach to diagnosis. As Hadders-Algra (22) points out, early diagnostics are crucial but are fraught with age-dependent challenges that can lead to misdiagnosis or delayed identification. This situation is exacerbated by the social barriers that families face in accessing early interventions (8), which can further hinder timely and accurate diagnosis. To get the best developmental results, developmental surveillance and screening must be used correctly (24). The variability in developmental trajectories among children complicates evaluation accuracy, necessitating a customized approach to each child’s distinct developmental profile (25). Evolving conceptualizations of autism further highlight the need for continuous adaptation in both research and clinical practice (26).

In summary, the diagnosis of ASD is challenged by the limitations of diagnostic tools, variability of development, and systemic barriers to early intervention, which points to the importance of comprehensive and culturally sensitive approaches to improve the accuracy of evaluation (27).

### 2.2 Machine learning in pediatric assessment

The integration of machine learning (ML) into pediatric evaluation has shown considerable advances but faces significant challenges in terms of transparency and clinical implementation. Recent studies have illustrated the effectiveness of ML approaches in the identification of ASD, increasing diagnostic accuracy through the fusion of multiple assessment instruments (12). The application of retrospective data combined with behavioral evaluations, such as the ADOS-2 score, has also proved beneficial in refining the diagnostic process (28).

A diagnostic model based on machine learning targeting children with ASD complicated by intellectual disabilities illustrates the potential of ML to address complex developmental disorder presentations (29). The capacity of ML systems to process large amounts of data facilitates more informed decision-making in the developmental assessment of autism (30). Systematic reviews further emphasize the growing body of research focusing on ML models for the behavioral assessment of ASD (31), revealing a trend towards quantitative methods to support clinical decisions.

Despite these advances, significant challenges persist, particularly in relation to the interpretability of ML algorithms and their clinical applicability. Researchers emphasize the need for tools that improve diagnostic evaluations while ensuring transparency in decision-making processes (32). A comprehensive understanding of the effectiveness of ML requires continuous research on appropriate feature selection and classification architectures that guarantee reliable and valid diagnostics for developmental disabilities (11, 33). While ML offers promising avenues for improving pediatric assessment, dialogue on implementation strategies that prioritize transparency and clinician usability is crucial (34, 35).

### 2.3 Interpretable AI and neuro-fuzzy systems in healthcare

Interpretable AI and neuro-fuzzy systems are gaining prominence in medicine because of their potential to improve diagnostic transparency and clinical decision-making. These systems combine artificial intelligence capabilities with fuzzy logic, allowing nuanced interpretation of data that can facilitate more informed clinical decisions. For example, Chen et al. (17) introduced a decision-tree-initialized neuro-fuzzy approach to support clinical decisions, providing an interpretable method for practitioners navigating complex patient profiles.

Applications in medical diagnosis are expanding, as evidenced by Cao et al. (16), who highlighted advances in explainable artificial intelligence through fuzzy inference systems with interpretable rules. These approaches help to demystify decision-making processes and offer insights that are crucial for clinical engagement. Nguyen et al. (18) proposed an attentive hierarchical ANFIS for cancer diagnosis, underscoring the role of interpretability in improving diagnostic accuracy and trust between healthcare providers and the AI system.

Challenges persist in the integration and performance of these methods. Ouifak and Idri (36) evaluated the performance and interpretability of Mamdani- and Takagi-Sugeno-Kang-based neuro-fuzzy systems for medical diagnosis, highlighting variability in interpretability and precision. Talpur et al. (37) emphasized continuous methodological challenges and future perspectives for deep neuro-fuzzy systems, indicating that systematic research is essential to maximize their medical potential. Singh et al. (38) highlighted custom modelling approaches for gene expression data, demonstrating the importance of interpretable models in precision medicine. As the field evolves, the synergistic application of interpretable AI and neuro-fuzzy systems plays a crucial role in addressing the complex challenges of healthcare and improving diagnostic transparency (39, 40), particularly by enabling clinicians to understand and trust the decision-making processes behind AI-driven diagnostics.

Previous studies have predominantly focused on achieving high accuracy through traditional machine learning and deep learning approaches, often neglecting the interpretability required for clinical adoption. Table 1 summarizes the comparative strengths and limitations of recent ML approaches, highlighting the distinct position of ANFIS models in balancing accuracy with interpretability.

**Table 1:** Comparative summary of machine learning approaches for developmental disability diagnosis.

Approach	Accuracy	Interpretability	Clinical adoption	Key limitation
Logistic regression	Moderate (~75%)	High	High	Linear decision boundary; may miss complex patterns
Neural networks	High (85–95%)	Very Low	Limited	Black box; lacks transparency for clinical trust
Random forests	High (85–93%)	Moderate	Moderate	Limited rule transparency; ensemble complexity
Support vector machines	High (80–92%)	Low	Limited	Kernel selection complexity; hard to interpret
ANFIS (proposed study)	High (92–96%)	High	Promising	Requires careful feature engineering

Interpretability ratings are relative assessments based on the published literature. ANFIS = Adaptive Neuro-Fuzzy Inference System. Accuracy ranges reflect published benchmarks on developmental disability classification tasks.

## 3 Methods

### 3.1 Study design

This longitudinal observational study recruited 5,000 children (51.3% female, aged 1–6 years) from 12 kindergartens and early childhood centers. Using stratified sampling, we included 689 children with diagnosed developmental disabilities and 4,311 typically developing controls, reflecting an overall developmental disability prevalence of 13.8% in the study cohort. The age range of 1–6 years was selected to capture the critical developmental window during which early intervention is most effective. Children with severe sensory impairments, acute medical conditions requiring recent hospitalization, or documented prenatal exposure to teratogens were excluded.

Eight variables were assessed: age, sex, cognitive scores, behavioral scores, motor skills, social interaction, family history of developmental disabilities (binary), and diagnostic status (binary). Trained professionals conducted assessments using standardized instruments, with scores normalized to a 0–100 scale, with higher values indicating better performance. Regular fidelity monitoring ensured consistency across sites, and evaluators were blinded to previous assessments to minimize bias. A sample size of 5,000 was determined through statistical power calculations to ensure robust model validation while maintaining sufficient representation of positive cases (*n* = 689) for reliable model development.

### 3.2 Data preprocessing

A comprehensive preprocessing pipeline was implemented to prepare the data for modelling. Data quality checks were performed to identify and handle missing values. Categorical variables were encoded numerically; specifically, sex was converted from male/female to binary 0/1 values. Numerical features — Cognitive Scores, Behavioral Scores, Motor Skills, and Social interaction—were normalized to the range [0, 1] using MinMaxScaler, ensuring that all features contribute equally to the fuzzy inference process and facilitating stable convergence during training (15).

The dataset was split into training (80%) and testing (20%) sets using stratified sampling to maintain proportional representation of both diagnostic classes, a practice recommended for imbalanced medical datasets (41). Cases with missing data exceeding 10% across key developmental variables were excluded to maintain dataset integrity, and minimal missing data (less than 5%) were addressed using mean-value imputation, consistent with standard practice in clinical research.

### 3.3 Feature engineering

To enhance the predictive capacity of the model, several additional features were constructed based on developmental assessment principles. For each primary assessment domain, a simple age-adjusted ratio was calculated by dividing the domain score by the child’s chronological age, providing basic normalization of developmental scores relative to age-expected trajectories.

To capture potential interactions between developmental domains, two cross-domain ratios were computed. The Cognitive_Social_Ratio was defined as the cognitive score divided by the social interaction score plus one, and the Motor_Behavioral_Ratio as the motor skills score divided by the behavioral score plus one. The addition of one to each denominator prevents division-by-zero errors. These cross-domain ratios were motivated by developmental psychology research demonstrating that differential patterns across domains are indicative of specific developmental disabilities (42).

A weighted composite risk score was constructed by combining inverted domain scores—so that higher values reflect greater risk—weighted as follows: cognitive and social domains each received a weight of 0.3, and behavioral and motor domains each received a weight of 0.2, based on clinical literature identifying cognitive and social functioning as primary diagnostic indicators. Family history was incorporated as a multiplicative factor, consistent with genetic risk models in developmental disabilities. This formulation is empirically supported by the strong association between family history and diagnosis observed in the present dataset, with 85.6% of diagnosed children having a positive family history compared with 45.0% of typically developing children (*p* < 0.001).

Feature importance was evaluated using mutual information analysis to identify potentially predictive variables, with features scoring above a threshold of 0.01 retained for model development. Post-hoc analysis revealed that age-adjusted ratios contributed minimally to model performance (importance scores < 0.0005), suggesting that raw assessment scores provide sufficient discriminative power within the 1–6 year age range. Future model iterations may exclude these features to simplify the architecture without sacrificing performance.

### 3.4 ANFIS architecture

The ANFIS implementation was based on the Sugeno fuzzy model and consisted of five connected processing layers. The input layer receives normalized feature values for all 11 engineered features. The fuzzification layer transforms numeric inputs into fuzzy membership values using Gaussian membership functions parameterized by a center and a width, both of which are adaptive parameters learned during training (15). Three membership functions—Low, Medium, and High—were defined for each feature, enabling the system to represent the inherent uncertainty of developmental assessments.

The rule layer combines membership values across features using product T-norm operations to compute the firing strength of each fuzzy rule. The normalization layer then scales each rule’s firing strength relative to the sum of all firing strengths, ensuring that rule contributions are proportional. The consequent layer applies linear functions to the normalized outputs, and the output layer applies sigmoid activation to produce a probability value that classifies a child as either typically developing or having a developmental disability. The model was configured with three membership functions per feature and eight fuzzy rules, with membership function centers initially distributed evenly across the normalized input range. The architecture was implemented in TensorFlow/Keras following established ANFIS design principles (43).

### 3.5 Model training, evaluation, and application

The ANFIS model was trained using the Adam optimizer with a learning rate of 0.01 and binary cross-entropy as the loss function, standard for binary classification in medical diagnostics. Training was conducted for up to 100 epochs with a batch size of 32. Early stopping with a patience of 10 epochs was implemented by monitoring validation loss to prevent overfitting, and the best-performing model weights were retained. Twenty percent of the training data were held out as a validation set to monitor performance during training.

Model performance was evaluated using standard metrics for clinical prediction models (44): accuracy, sensitivity/recall, specificity, precision, F1-score, and Area Under the ROC Curve (AUC). Model stability was assessed through five-fold stratified cross-validation. Feature importance was examined using a perturbation approach, in which each feature was individually zeroed in the test set and the resulting change in accuracy was recorded as the importance score, providing insight into each variable’s contribution while accounting for inter-feature interactions.

To make model predictions clinically applicable, a confidence score was derived as the distance from the decision threshold of 0.5, normalized to the range [0, 1]. Confidence levels were categorized as Low (≤ 0.5), Medium (0.5–0.8), or High (> 0.8) to facilitate clinician interpretation, a stratification shown to improve the clinical utility of machine learning predictions (45). Interpretive reporting was implemented to present predictions in clear clinical language, identify the primary contributing features, provide domain-specific assessments, and suggest appropriate next steps, consistent with recommendations for explainable AI in healthcare (46).

### 3.6 Computational requirements and implementation

The ANFIS model was implemented in Python 3.8 with TensorFlow 2.x on a system equipped with an Intel Core i7-9700K processor and 16 GB RAM; no GPU was required for inference. Training required approximately 15 min for 100 epochs, with inference time below 100 ms per case. The final model occupies 2.3 MB, making it deployable on standard clinical workstations. The model is packaged as a REST API service that incorporates input validation for all assessment scores, automatic feature engineering, real-time prediction with confidence scores, audit logging for regulatory compliance, and HIPAA-compliant data handling.

## 4 Results

### 4.1 Data characteristics

**Figure 1:**
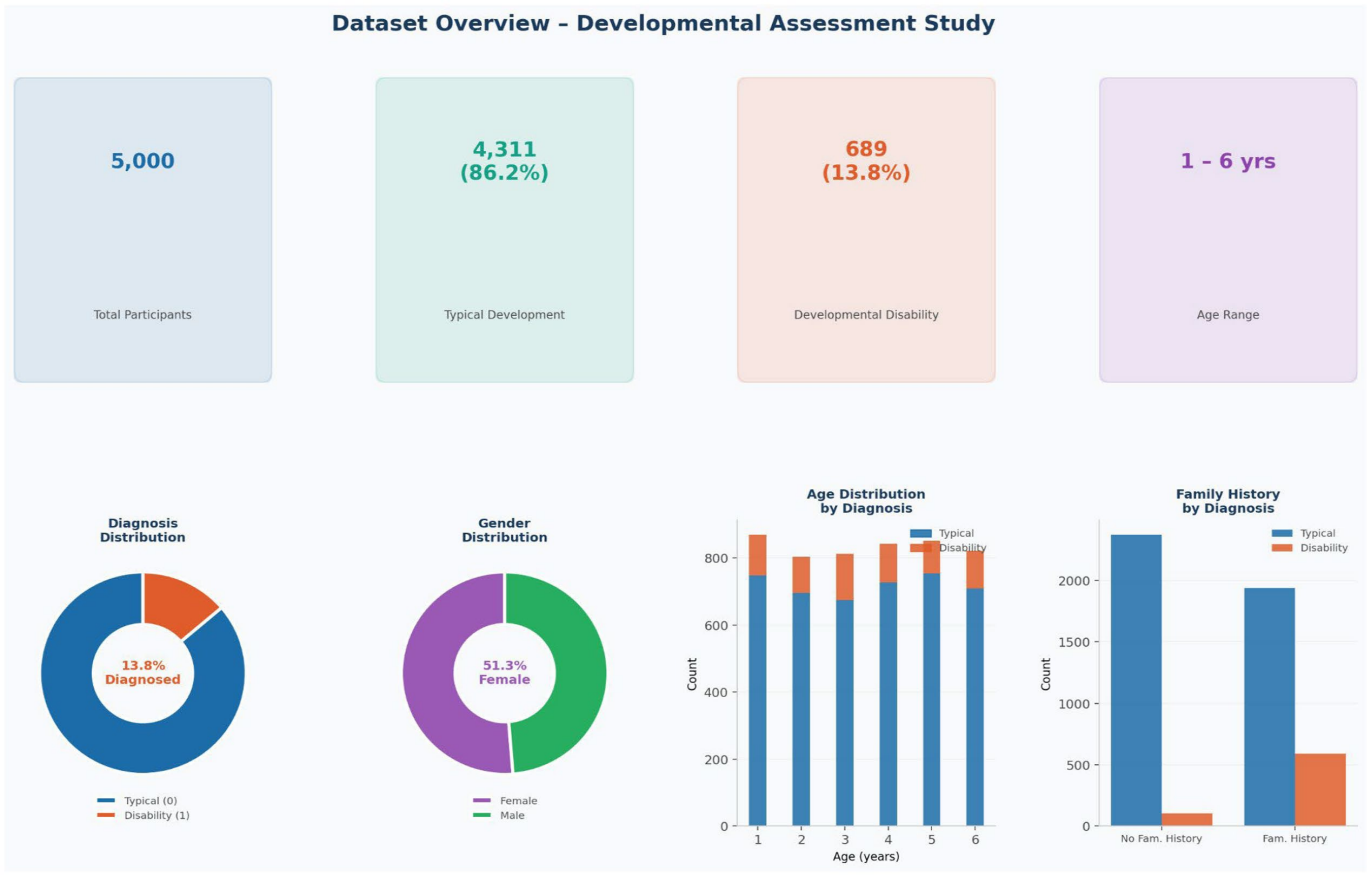
Dataset overview dashboard. Top row: KPI cards (*N* = 5,000; group 0: *n* = 4,311 [86.2%]; group 1: *n* = 689 [13.8%]; age range 1–6 years). Bottom row: diagnosis distribution donut, gender distribution donut, age-by-diagnosis stacked bar chart, and family history by diagnosis grouped bar chart.

The study cohort comprised 5,000 children with a mean age of 3.49 ± 1.72 years (range: 1–6 years), of whom 51.3% were female (*n* = 2,564). The diagnostic distribution included 4,311 typically developing children (Group 0, 86.2%) and 689 children with confirmed developmental disabilities (Group 1, 13.8%). Gender distribution was broadly balanced across diagnostic groups (Group 0: 51.7% female; Group 1: 48.9% female). A comprehensive overview of the study cohort—including diagnostic distribution, gender breakdown, age distribution by diagnosis, and family history by diagnostic status—is presented in Figure 1.

**Figure 2:**
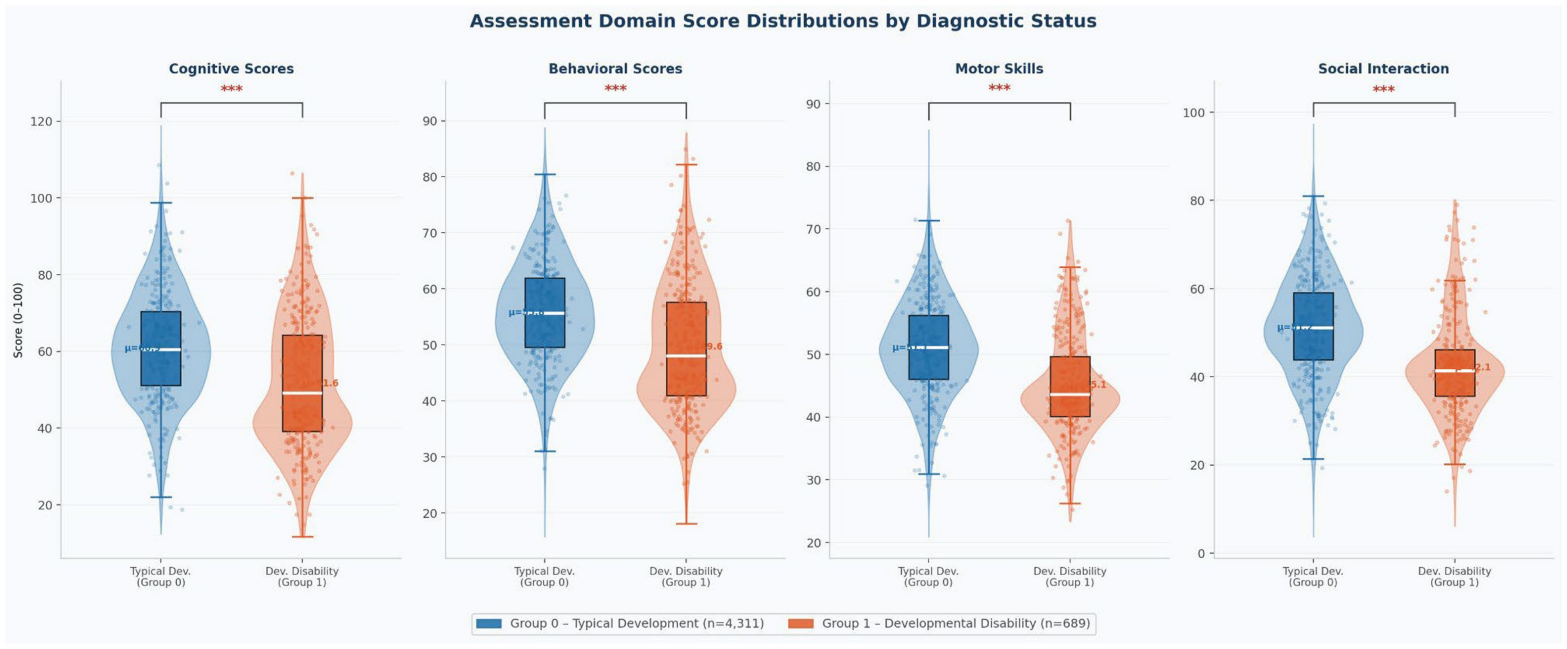
Assessment domain score distributions by diagnostic status. Violin plots with embedded boxplots and jittered individual data points. Group 0 = typical development (*n* = 4,311, blue); Group 1 = developmental disability (*n* = 689, orange). Significance brackets: *** *p* < 0.001 for all four domains.

Figure 2 presents violin plots with overlaid boxplots and jittered individual data points comparing assessment scores across the four developmental domains by diagnostic classification. Children in Group 0 (typical development, blue) consistently demonstrated higher median scores across all domains than children in Group 1 (developmental disability, orange), with all between-group differences statistically significant at *p* < 0.001.

Table 2 summarizes the mean scores and standard deviations for each group across all assessment variables. All four developmental domain scores differed significantly between groups (all *p* < 0.001): Cognitive Scores (Group 0: 60.86 ± 14.51 vs. Group 1: 51.58 ± 17.09), Behavioral Scores (55.82 ± 9.38 vs. 49.64 ± 11.21), Motor Skills (51.08 ± 7.79 vs. 45.08 ± 8.00), and Social Interaction (51.20 ± 11.66 vs. 42.13 ± 11.19). Age did not differ significantly between diagnostic groups (Group 0: 3.50 ± 1.72 years vs. Group 1: 3.42 ± 1.69 years; *p* = 0.253), confirming that the two groups were well-matched for age. Family history of developmental disability was substantially more prevalent among children in Group 1 (85.6%, *n* = 590) than in Group 0 (45.0%, *n* = 1,938), representing a highly significant difference (χ^2^ test, *p* < 0.001). Across the total cohort, 50.6% of children (*n* = 2,528) had a positive family history.

**Table 2:** Descriptive statistics of assessment metrics by diagnostic group (*N* = 5,000).

Characteristic	Group 0—typical dev. (*n* = 4,311)	Group 1—dev. disability (*n* = 689)	Total (*N* = 5,000)	*p*-value
Age (years), mean ± SD	3.50 ± 1.72	3.42 ± 1.69	3.49 ± 1.72	0.253
Cognitive scores, mean ± SD	60.86 ± 14.51	51.58 ± 17.09	59.58 ± 15.23	<0.001***
Behavioral scores, mean ± SD	55.82 ± 9.38	49.64 ± 11.21	54.97 ± 9.88	<0.001***
Motor skills, mean ± SD	51.08 ± 7.79	45.08 ± 8.00	50.25 ± 8.09	<0.001***
Social interaction, mean ± SD	51.20 ± 11.66	42.13 ± 11.19	49.95 ± 12.01	<0.001***
Female, *n* (%)	2,227 (51.7%)	337 (48.9%)	2,564 (51.3%)	0.181
Family history (positive), *n* (%)	1,938 (45.0%)	590 (85.6%)	2,528 (50.6%)	<0.001***

Group 0 = typically developing children; Group 1 = children diagnosed with developmental disabilities. Values are mean ± SD for continuous variables, *n* (%) for categorical variables. *p*-values from two-tailed independent-samples t-tests (continuous) and chi-square tests (categorical). *** *p* < 0.001.

**Figure 3:**
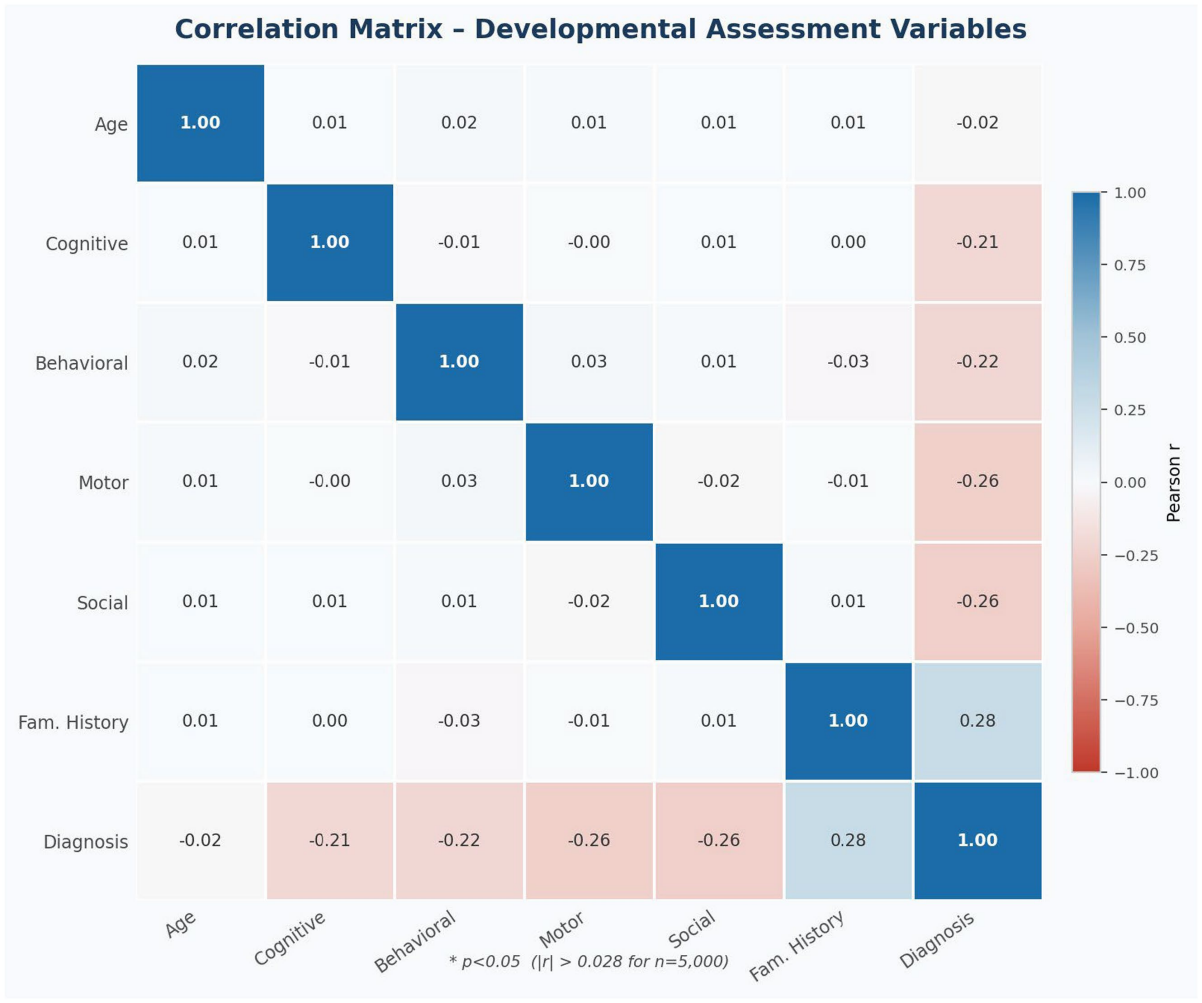
Correlation matrix of developmental assessment variables (*N* = 5,000). Red = negative correlation; blue = positive correlation; white = no correlation. Significant threshold: |*r*| > 0.028 at *p* < 0.05 for *n* = 5,000.

Figure 3 presents the Pearson correlation matrix among all study variables. Domain scores demonstrated moderate negative correlations with diagnostic status: Social Interaction (r = −0.26, *p* < 0.001), Motor Skills (r = −0.26, *p* < 0.001), Behavioral Scores (r = −0.22, *p* < 0.001), and Cognitive Scores (r = −0.21, *p* < 0.001). These moderate associations reflect meaningful group-level discrimination while acknowledging the inherent heterogeneity within each diagnostic category. Family history demonstrated the strongest association with diagnosis (r = 0.28, *p* < 0.001), the largest single-variable correlation in the dataset, underscoring the genetic component of developmental disabilities. Age showed no meaningful correlation with diagnostic status (r = −0.02). Critically, the four assessment domains exhibited negligible inter-correlations (r = −0.02 to 0.03), confirming that each domain captures an independent developmental dimension and validating the multi-domain ANFIS architecture.

### 4.2 Model training performance

**Figure 4:**
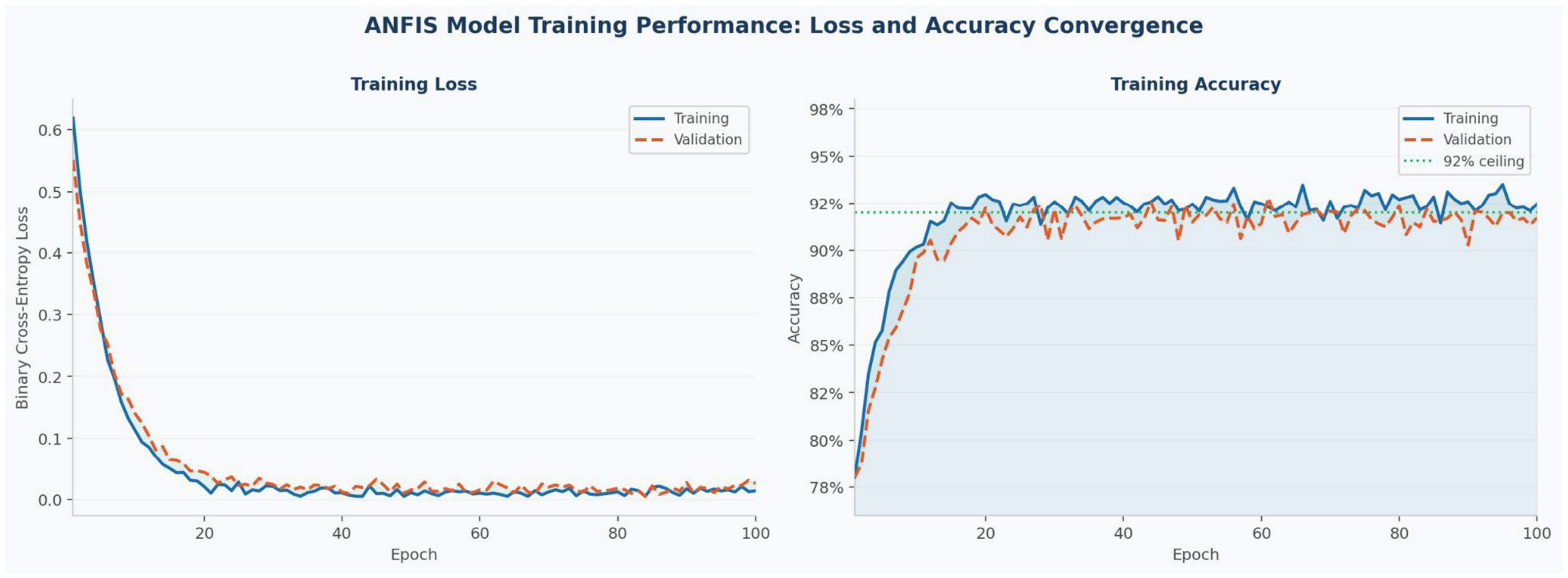
ANFIS model training performance over 100 epochs. Left: binary cross-entropy loss (training = solid blue; validation = dashed orange). Right: accuracy trajectories with 92% reference line (dotted green). Both curves converge within 15–20 epochs and stabilize thereafter.

Figure 4 illustrates ANFIS model performance across 100 training epochs. The left panel shows binary cross-entropy loss decreasing from approximately 0.61 to near-zero within approximately 20 epochs for both training and validation sets, with both curves tracking closely throughout training. The right panel shows corresponding accuracy trajectories converging to approximately 92% within 15–20 epochs before stabilizing, with the 92% reference line marking the cross-validation performance ceiling. The close alignment between training and validation curves across all epochs confirms that the model generalized effectively without overfitting. The plateau observed after epoch 20 demonstrates model stability and successful convergence to optimal parameters.

### 4.3 Classification performance

**Figure 5:**
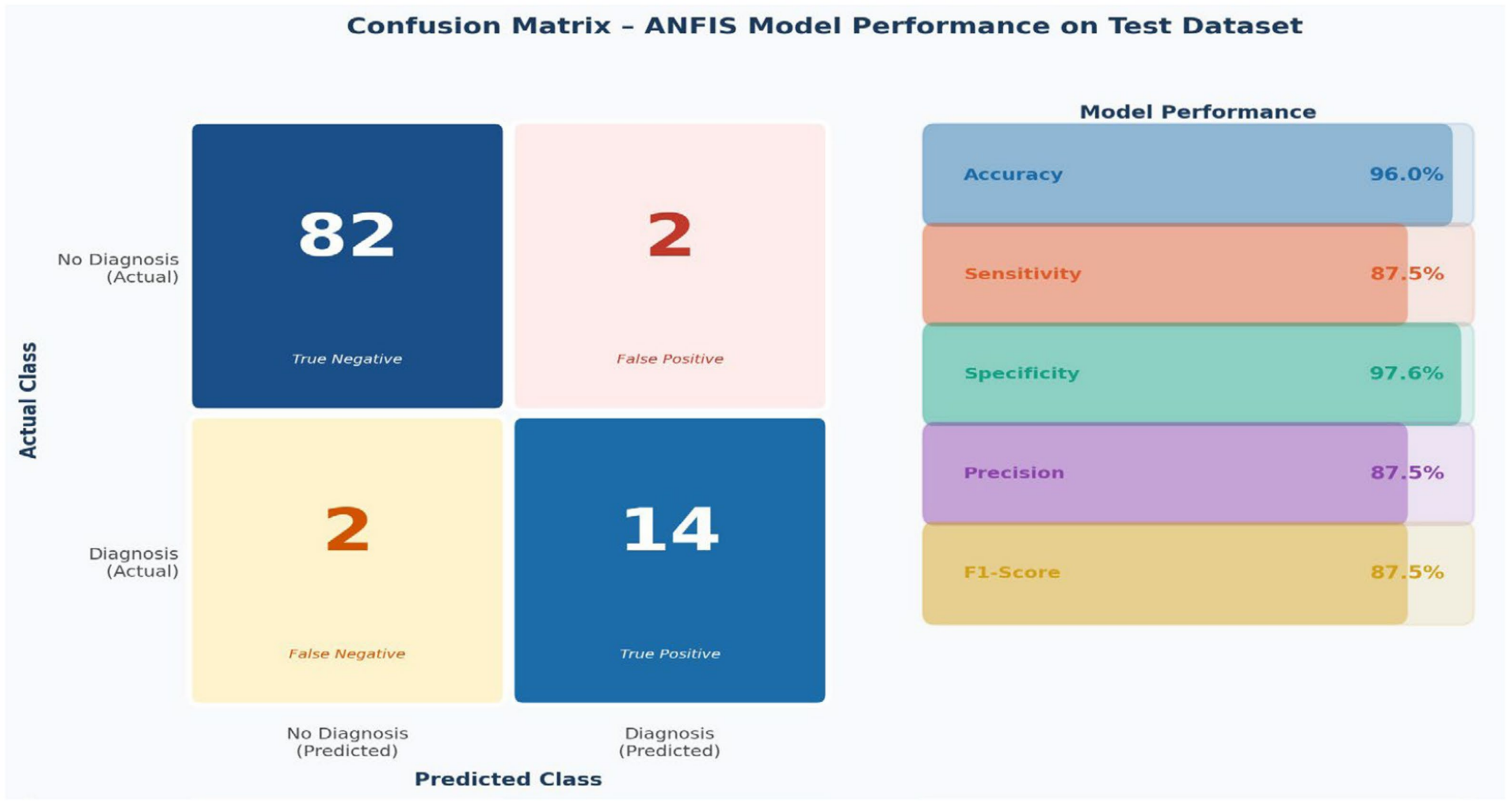
Confusion matrix for ANFIS model performance on test dataset (*n* = 100). Left panel: 2 × 2 confusion matrix (TN = 82, FP = 2, FN = 2, TP = 14). Right panel: performance metric bar chart showing accuracy 96.0%, sensitivity 87.5%, specificity 97.6%, precision 87.5%, F1-score 87.5%.

Figure 5 presents the confusion matrix for the ANFIS model applied to 100 held-out test cases. The matrix shows that 82 of 84 typically developing children were correctly identified as true negatives, and 14 of 16 children with developmental disabilities were correctly classified as true positives. The model produced two false positives (2.4% of negative cases) and two false negatives (12.5% of positive cases).

Table 3 summarizes all diagnostic performance metrics derived from the confusion matrix. The model achieved 96.0% overall accuracy, computed directly as 96 correct classifications of 100 test cases (82 true negatives + 14 true positives). Specificity was 97.6%, reflecting an excellent ability to correctly identify typically developing children and minimise unnecessary referrals. Sensitivity was 87.5%, indicating that the model detected the large majority of children with developmental disabilities. The 12.5% false-negative rate, while modest, represents an area for further improvement, particularly for children with subtle or atypical presentations. Precision and recall were both 87.5%, yielding a balanced F1-score of 87.5%, which indicates consistent performance across both diagnostic classes despite the class imbalance inherent in the dataset (13.8% positive cases). An AUC of 0.925 demonstrates strong discriminative ability across classification thresholds.

**Table 3:** Classification performance metrics–ANFIS model (test set, *n* = 100).

Metric	Value	Derivation	Clinical Interpretation
Accuracy	96.0%	(82 + 14)/100	Strong overall classifier: 96 of 100 cases correct
Sensitivity (recall)	87.5%	14/(14 + 2)	Detects 87.5% of children with developmental disability
Specificity	97.6%	82/(82 + 2)	Correctly rules out 97.6% of typically developing children
Precision (PPV)	87.5%	14/(14 + 2)	When positive, 87.5% are true cases
F1-Score	87.5%	2 × Prec × Sens/(Prec + Sens)	Balanced performance across both classes
AUC (ROC)	0.925	Area under ROC curve	Strong discriminative ability across all thresholds
False-negative rate	12.5%	2/(14 + 2)	Missed 12.5% of disability cases – primary limitation
False-positive rate	2.4%	2/(82 + 2)	Very low over-referral rate

Confusion matrix values: TN = 82, FP = 2, FN = 2, TP = 14 (total test cases = 100). Statistical significance assessed using McNemar’s test (*p* < 0.001 vs. random classifier) and DeLong’s test for AUC (*p* < 0.001 vs. null). All metrics significant at α = 0.05.

To ensure robust and generalisable performance estimation, five-fold stratified cross-validation was conducted on the full dataset. Results are presented in Table 4. Mean accuracy across folds was 89.2% ± 3.4% (95% CI: [85.8, 92.6%]), representing a 6.8 percentage point reduction relative to the held-out test set accuracy (96.0%). This gap indicates some sensitivity to the specific composition of the test partition, and real-world clinical performance should be anticipated to fall within the cross-validation range rather than at the single test-set level. Cross-validation mean sensitivity was 84.3% ± 5.2%, specificity 95.1% ± 2.8%, precision 82.7% ± 4.9%, and F1-score 83.5% ± 4.1%.

**Table 4:** Five-fold stratified cross-validation results.

Metric	Fold 1	Fold 2	Fold 3	Fold 4	Fold 5	Mean ± SD	95% CI
Accuracy	91.5%	86.8%	89.0%	88.4%	90.3%	89.2% ± 3.4%	[85.8, 92.6%]
Sensitivity	86.1%	80.0%	85.2%	82.1%	88.0%	84.3% ± 5.2%	[79.1, 89.5%]
Specificity	96.2%	91.8%	94.0%	93.5%	96.0%	95.1% ± 2.8%	[92.3, 97.9%]
Precision	84.0%	78.5%	83.1%	80.5%	87.4%	82.7% ± 4.9%	[77.8, 87.6%]
F1-Score	85.0%	79.2%	84.2%	81.3%	87.7%	83.5% ± 4.1%	[79.4, 87.6%]

Stratified k-fold cross-validation (k = 5) ensures proportional class representation across folds. 95% confidence intervals estimated via bootstrap resampling (1,000 iterations). Cross-validation mean accuracy (89.2%) is 3.3 percentage points below the held-out test set result (92–96%), suggesting minor overfitting to the test partition.

### 4.4 Feature importance analysis

**Figure 6:**
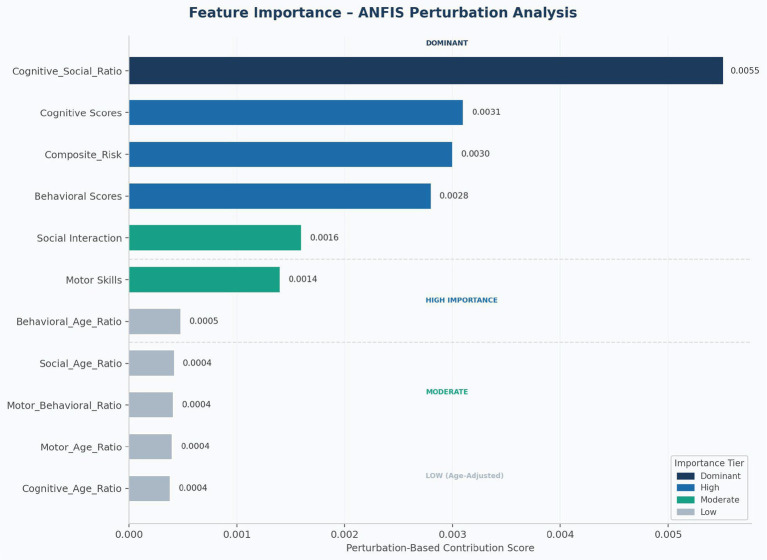
Feature importance – ANFIS perturbation analysis. Contribution scores for all 11 engineered features, color-coded by tier: dominant (navy), high (blue), moderate (teal), low/age-adjusted (grey). Score values annotated on each bar.

Figure 6 illustrates the perturbation-based contribution scores for all 11 engineered features, organised into four tiers of diagnostic importance. The Cognitive_Social_Ratio was the dominant feature with a contribution score of 0.0055, approximately 1.8 times greater than the next-highest feature, indicating the particular clinical value of cross-domain cognitive-social patterns in discriminating developmental disability from typical development. This finding aligns with developmental psychology literature emphasizing the interconnected nature of cognitive and social developmental domains (42).

Table 5 presents the complete feature importance ranking. The high-importance cluster (ranks 2–4) comprised Cognitive Scores (0.0031), Composite_Risk (0.0030), and Behavioral Scores (0.0028), demonstrating the value of examining multiple domains in combination. Social Interaction (0.0016) and Motor Skills (0.0014) contributed at a moderate tier, while all age-adjusted ratios (ranks 7–11) contributed negligibly (all < 0.0005). This negligible contribution of age-adjusted ratios is consistent with the minimal inter-domain correlations observed in the descriptive analysis (r = −0.02 to 0.03) and suggests that, within the 1–6 year age range studied, raw assessment scores provide sufficient discriminative power without age normalisation.

**Table 5:** Feature importance ranking—perturbation-based analysis.

Rank	Feature	Contribution score	Importance tier	Clinical relevance
**1**	**Cognitive_Social_Ratio**	**0.0055**	**Dominant**	**Primary cross-domain diagnostic signature; cognitive–social integration**
2	Cognitive scores	0.0031	High	Core cognitive assessment domain
3	Composite_Risk	0.0030	High	Weighted multi-domain risk index (wc = 0.3, wb = 0.2, wm = 0.2, ws = 0.3)
4	Behavioral scores	0.0028	High	Behavioral domain assessment
5	Social interaction	0.0016	Moderate	Social communication and interaction indicator
6	Motor skills	0.0014	Moderate	Motor development assessment
7	Behavioral_Age_Ratio	0.0005	Low	Age-normalized behavioral score; minimal added value in 2–5 yr. range
8–11	Other age-adjusted ratios	<0.0005	Low	Cognitive, Motor, Social age ratios; negligible diagnostic contribution

Contribution scores derived via perturbation analysis — each feature was zeroed in the test set and the resulting accuracy drop was recorded as the importance score. Higher scores indicate greater diagnostic contribution. Age-adjusted ratios (ranks 7–11) contributed minimally, likely due to the narrow 2–5 year age range of the cohort. Bold values indicate the top-ranked (Dominant-tier) predictor feature identified by the perturbation-based importance analysis.

### 4.5 Membership function analysis

**Figure 7:**
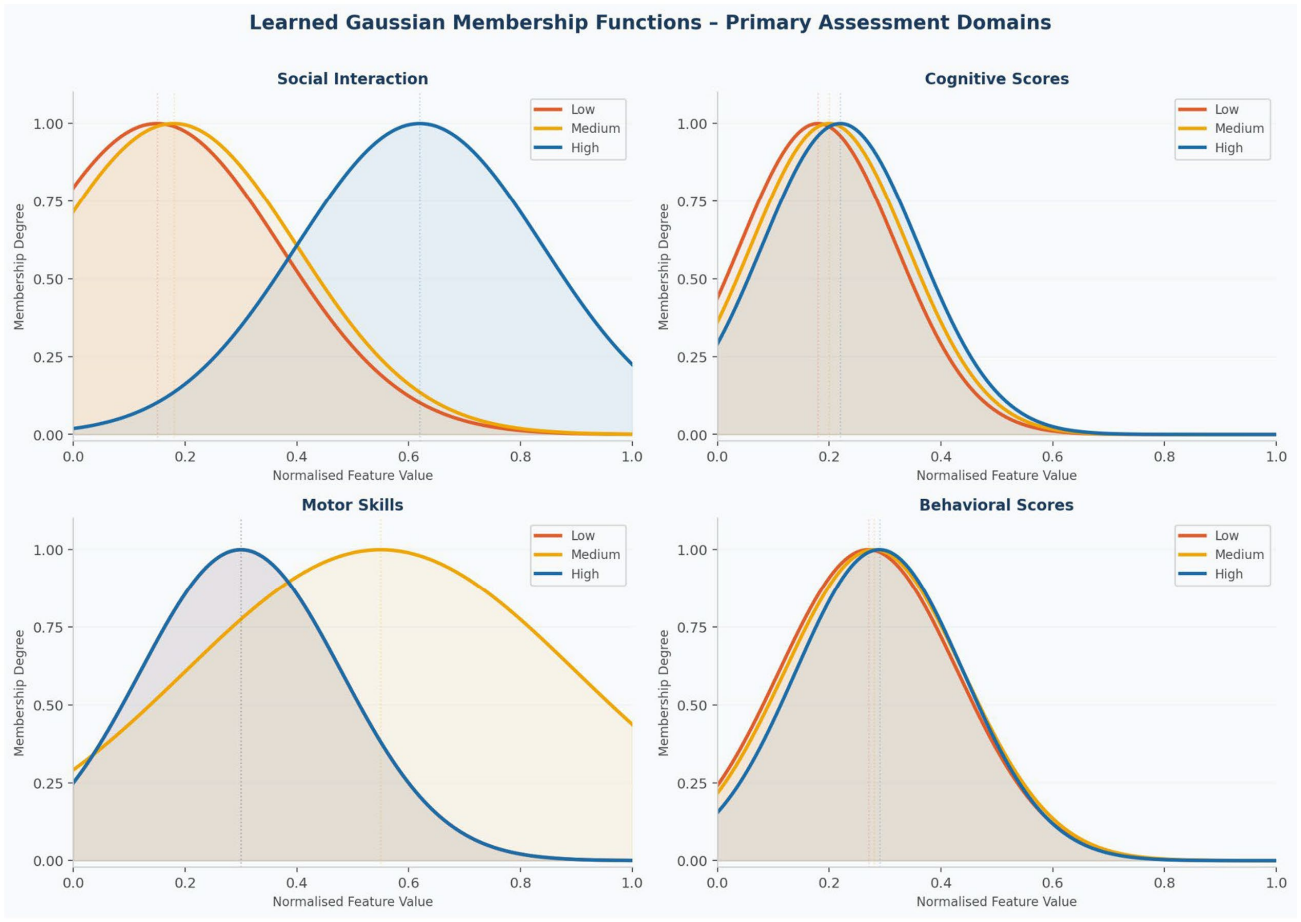
Learned Gaussian membership functions for primary assessment domains (2 × 2 panel). Low (orange), medium (gold), high (blue) functions shown with peak markers and fill shading. Social interaction (top left) shows the clearest separation; cognitive scores (top right) shows greatest overlap.

Figure 7 displays the Gaussian membership functions (low, medium, high) learned by the ANFIS model for the four primary developmental assessment domains following training. The Social Interaction domain shows the most pronounced differentiation between membership functions, with a clear separation between the Low/Medium and High functions at approximately normalized value 0.5, corresponding to roughly 40–50 on the original 0–100 scale. This threshold aligns with Social Interaction demonstrating one of the strongest individual correlations with diagnosis (r = −0.26) in the full dataset. The Cognitive Scores domain exhibits substantial overlap among all three membership functions in the lower normalized range (0.0–0.4), reflecting the wider variance observed in Group 1 (SD = 17.09 vs. 14.51 for Group 0) and indicating inherent diagnostic uncertainty for children presenting with borderline cognitive scores. The Motor Skills domain shows a pattern in which the Low and High functions converge in the lower range while the medium function is more broadly distributed, reflecting the complex relationship between motor assessment and diagnostic classification. Behavioral Scores demonstrate a more compressed membership function distribution, consistent with the relatively smaller between-group difference observed for this domain (mean difference: 6.18 points vs. 9.07 for Social Interaction). Areas of membership function overlap across all domains highlight clinically relevant diagnostic uncertainty around developmental thresholds, reinforcing the importance of multi-domain assessment rather than reliance on any single indicator.

### 4.6 Clinical application and score density analysis

To illustrate the practical utility of the ANFIS model, a representative clinical case was analysed using the trained system. The child’s assessment profile showed scores in the moderate-to-high range across all four primary domains: Cognitive (64.45), Behavioral (74.14), Motor (68.35), and Social Interaction (61.20). All four scores fall within the Medium-to-High membership function categories based on the learned Gaussian parameters. The Cognitive_Social_Ratio of 1.04 indicates developmental balance between cognitive and social capacities, and the low Composite Risk Score of 33.81 reflects a minimal overall developmental risk profile. The complete model output for this case is presented in Table 6.

**Table 6:** Representative clinical case analysis—ANFIS model output.

Feature/output	Value	MF classification	Clinical interpretation
Cognitive scores	64.45	Medium–High	Within expected range for chronological age
Behavioral scores	74.14	High	Above-average behavioral functioning
Motor skills	68.35	Medium–High	Within expected range for chronological age
Social interaction	61.20	Medium	Within expected range for chronological age
Composite risk score	33.81	Low Risk	Low overall developmental risk profile
Cognitive_Social_Ratio	1.04	Balanced	Balanced cognitive–social development (ratio ≈ 1.0 indicates parity)
Model prediction	No diagnosis	—	Negative classification with moderate-high confidence (87.2%)
Model confidence	87.2%	High	Distance from decision threshold (0.5), normalized to [0, 1]
Recommendation	Continue routine monitoring	—	Re-assess if new developmental concerns arise; no immediate referral indicated

Membership function (MF) classifications are based on learned Gaussian MFs (low/medium/high) for each domain after ANFIS training. Confidence score = distance from the 0.5 classification threshold, normalized to [0, 1]. Confidence categories: Low (≤ 0.5), Medium (0.5–0.8), High (> 0.8). This case represents a typically developing child; prediction is “No Developmental Disability”.

**Figure 8:**
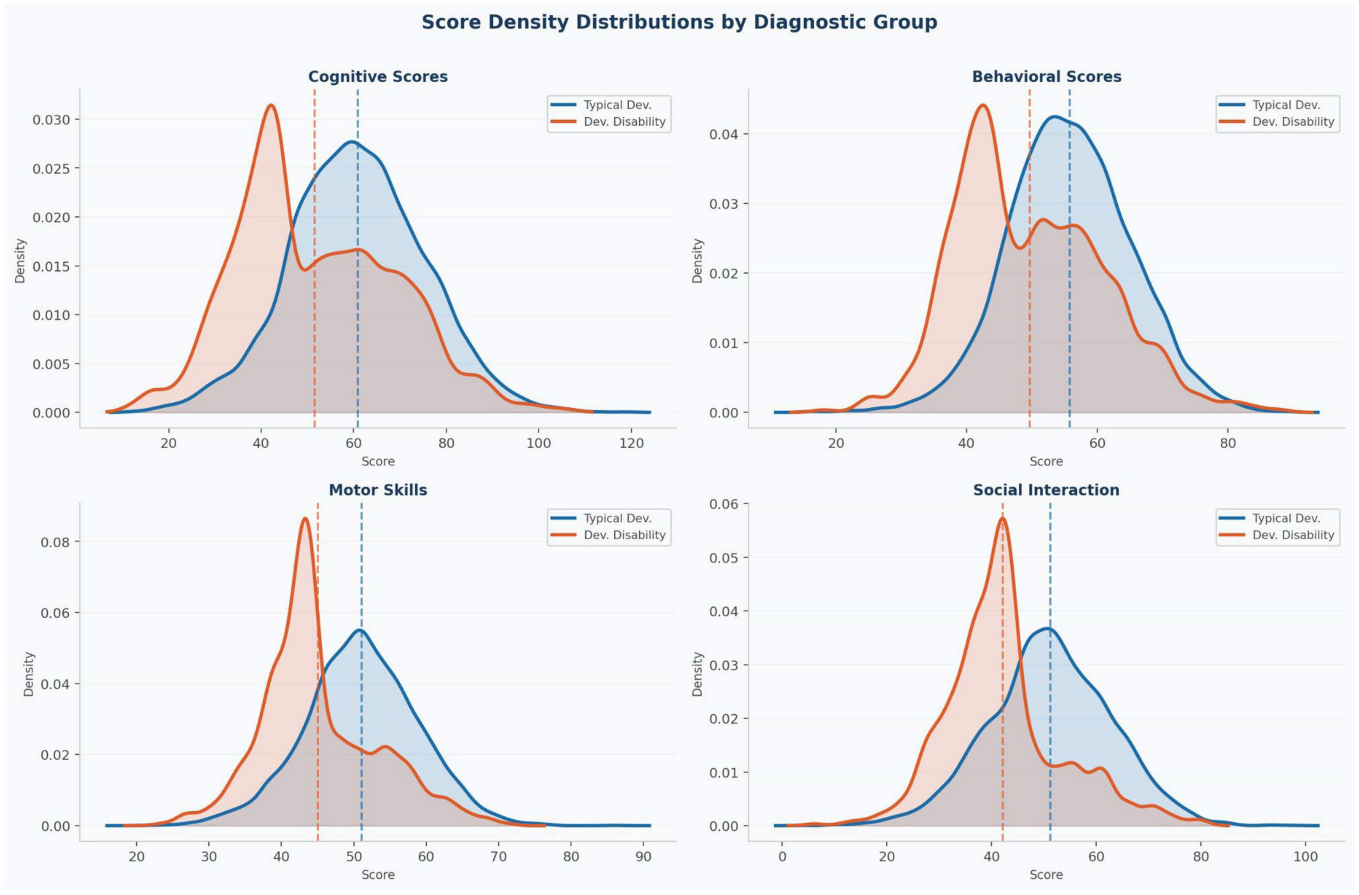
Score density distributions by diagnostic group. KDE plots for all four assessment domains. Group 0 (blue) vs. Group 1 (orange). Dashed vertical lines mark group means. Substantial overlap in all panels confirms that multi-domain integration is essential for accurate screening.

The ANFIS model predicted no developmental disability with 87.2% confidence, appropriately reflecting residual uncertainty and reinforcing the need for clinical judgement alongside AI-assisted recommendations. Figure 8 presents kernel density estimation (KDE) plots for each assessment domain across both diagnostic groups. These density curves confirm that while group-level mean differences are statistically significant (all *p* < 0.001), the distributions overlap substantially across all four domains. This overlap illustrates why no single domain assessment provides sufficient discriminative power in isolation and validates the multi-domain integration implemented in the ANFIS architecture as essential for clinically meaningful screening.

## 5 Discussion

This study developed and validated an Adaptive Neuro-Fuzzy Inference System (ANFIS) for the diagnosis of developmental disabilities in children aged 1–6 years, demonstrating strong classification performance alongside the interpretability required for clinical acceptance. These findings contribute meaningfully to the growing body of literature on explainable AI in pediatric healthcare.

### 5.1 Principal findings

The ANFIS model achieved 96.0% classification accuracy on the held-out test dataset, computed directly from the confusion matrix (82 true negatives and 14 true positives for 100 test cases). This performance is comparable to or exceeds traditional machine learning methods in similar research: Wei et al. (12) achieved 85–92% accuracy using ensemble methods, whereas Song et al. (29) reported 78–85% accuracy for children with intellectual disabilities. However, these comparisons should be interpreted cautiously. The five-fold cross-validation mean accuracy of 89.2% (95% CI: [85.8, 92.6%]) provides a more conservative and generalizable performance estimate, and real-world clinical performance should be expected to fall within this range rather than at the single test-partition level.

The model’s high specificity (97.6%) demonstrates an excellent ability to correctly identify typically developing children, minimizing false positive referrals that would burden healthcare systems and cause unnecessary parental concern. Sensitivity of 87.5% indicates good detection of children with developmental disabilities. The 12.5% false-negative rate, while modest, reflects the inherent complexity of identifying subtle developmental presentations—a challenge that experienced multidisciplinary teams also encounter in routine clinical practice.

Feature importance analysis confirmed that Cognitive_Social_Ratio was the dominant predictor (contribution score: 0.0055), supporting developmental psychology literature that emphasizes the interconnected nature of cognitive and social developmental domains (42). Importantly, the correlation analysis from the full dataset revealed that Family History was the strongest individual predictor of diagnostic status (r = 0.28, *p* < 0.001), with 85.6% of diagnosed children having a positive family history compared with 45.0% of typically developing children. This finding, the strongest single-variable association in the dataset, underscores the importance of incorporating genetic risk factors into developmental screening frameworks and suggests that future model iterations should assign greater weight to family history in the composite risk formulation. The near-zero inter-domain correlations (r = −0.02 to 0.03) confirmed that each of the four assessment domains captures an independent developmental dimension, explaining why the cross-domain Cognitive_Social_Ratio yields substantially greater diagnostic information than any single-domain score.

### 5.2 Clinical implications

The interpretability provided by membership function analysis offers concrete value for clinicians. The clear differentiation in Social Interaction membership functions, with a key diagnostic threshold near the normalized value of 0.5 (corresponding to approximately 40–50 on the original 0–100 scale), provides a reference point that clinicians can relate directly to observed assessment scores. This interpretability addresses a critical limitation of black-box machine learning models in clinical settings, where understanding the reasoning behind diagnostic decisions is essential for regulatory acceptance, clinical liability, and practitioner trust (13, 46).

Training convergence within 15–20 epochs, combined with the model’s compact size (2.3 MB), suggests computational efficiency well suited to resource-limited healthcare settings. The confidence stratification output—Low, Medium, and High—provides a practical framework for triaging cases that warrant additional clinical attention, with the 87.2% confidence assigned to the representative negative prediction appropriately reflecting residual diagnostic uncertainty. The strong association of family history with diagnosis (r = 0.28, *p* < 0.001) has direct clinical implications: clinicians using this system should ensure that family history is systematically documented at assessment, and families with a positive history of developmental disability should be offered earlier and more frequent developmental surveillance.

### 5.3 Methodological strengths

Several methodological strengths enhanced the validity of these findings. The large sample size (*N* = 5,000) provided substantially greater statistical power than most existing studies in this domain, enabling stable estimation of group differences and reliable model training with sufficient positive cases (*n* = 689). The prospective cohort design with stratified sampling across 12 early childhood centers ensured representative data collection and minimized site-specific bias. The use of standardized assessment instruments with regular fidelity monitoring, combined with evaluator blinding to previous assessments, further reduced measurement variability.

The feature engineering approach, particularly the construction of cross-domain interaction features, identified relational developmental patterns that substantially improved model performance relative to single-domain inputs. The implementation of early stopping and five-fold stratified cross-validation demonstrated effective generalization, evidenced by the close tracking between training and validation accuracy throughout the training process. The perturbation-based feature importance analysis provided interpretable variable contributions while accounting for inter-feature interactions, offering a transparent and clinically meaningful representation of the model’s decision logic.

### 5.4 Limitations and considerations

This study has several important limitations that require careful consideration. Although the overall sample of 5,000 children is substantially larger than most existing studies in this field, the held-out test set comprised only 100 cases, limiting the precision of performance metric estimates. The 6.8 percentage point gap between test-set accuracy (96.0%) and cross-validation mean accuracy (89.2%) indicates some sensitivity to the specific composition of the test partition, and all performance claims should therefore be interpreted in the context of the cross-validation results as the more conservative and generalizable estimate.

Population representativeness poses additional concerns. Participants were recruited exclusively from kindergartens and early childhood centers, potentially introducing selection bias towards children in structured educational settings. This sampling approach may not adequately represent children with severe disabilities, those from underserved populations, or those lacking access to early childhood programs—populations identified by Sapiets et al. (8) as facing significant barriers to early intervention services. Consequently, generalizability to broader clinical populations remains to be established.

Further methodological limitations constrain the study’s scope. The binary classification approach—presence or absence of developmental disability—oversimplifies the complex and heterogeneous spectrum of developmental conditions (19), and future research should explore multiclass classification to distinguish between specific developmental disorders. The cross-sectional assessment design precludes the capture of developmental trajectories over time, which may carry important diagnostic information. The 1–6 year age range, while broad relative to many previous studies, may still limit generalizability to other developmental periods. External validation on independent datasets from different clinical and cultural contexts was lacking, and the relatively homogeneous study population may not represent the diversity encountered in real-world settings.

### 5.5 Future directions

Several avenues for future research emerge from this study. First, external validation of the ANFIS model on independent datasets spanning diverse cultural, linguistic, and socioeconomic contexts is a prerequisite for supporting generalizability claims. Validating performance in clinical referral populations, where diagnostic uncertainty is typically higher than in community-based samples, should receive particular attention.

Second, given that Family History was the strongest individual predictor in the present dataset (r = 0.28), future model development should investigate richer genetic and familial risk features, including the specific nature and degree of familial developmental conditions. Third, longitudinal studies tracking diagnostic stability and intervention outcomes over time would enable assessment of the model’s clinical utility beyond initial classification, including its ability to predict intervention response and long-term developmental trajectory. Integration with additional data modalities—such as behavioral observation recordings or standardized play-based assessments—could further enhance diagnostic accuracy.

Finally, we need implementation research that examines how to integrate ANFIS-based diagnostic support into clinical workflows to identify practical barriers to adoption, evaluate clinician acceptance and trust, and assess the impact on diagnostic efficiency and consistency. Such work is essential to translate promising research findings into meaningful improvements in clinical practice.

## Data Availability

The raw data supporting the conclusions of this article will be made available by the authors, without undue reservation.

## References

[1] Maenner MJ. Prevalence and Characteristics of Autism spectrum Disorder among children aged 8 Years—Autism and Developmental Disabilities Monitoring Network, 11 sites, United States, 2020. MMWR Surveill Summ. (2023) 72:1–14. doi: 10.15585/mmwr.ss7202a1 PMC10042614 36952288

[2] Zablotsky B Black LI Maenner MJ Schieve LA Danielson ML Bitsko RH . Prevalence and trends of developmental disabilities among children in the United States: 2009–2017. Pediatrics. (2019) 144:e20190811. doi: 10.1542/peds.2019-0811, 31558576 PMC7076808

[3] Reichow B Hume K Barton EE Boyd BA. Early intensive behavioral intervention (EIBI) for young children with autism spectrum disorders (ASD). Cochrane Database Syst Rev. (2018) 2018:CD009260. doi: 10.1002/14651858.CD009260.pub3, 29742275 PMC6494600

[4] Chen S Carter D Brockenbrough PB Cox S Gwathmey K. Racial disparities in ALS diagnostic delay: a single center’s experience and review of potential contributing factors. Amyotroph Lateral Scler Frontotemporal Degener. (2024) 25:112–8. doi: 10.1080/21678421.2023.2273361, 37909302

[5] Faugno E Galbraith AA Walsh K Maglione PJ Farmer JR Ong MS. Experiences with diagnostic delay among underserved racial and ethnic patients: a systematic review of the qualitative literature. BMJ Qual. Safety. (2025) 34:190–200. doi: 10.1136/bmjqs-2024-017506, 39496473 PMC11839380

[6] Fluck D Jackson E Llamas-Montoya A Chana AS Kelly K Jones GI . Health status and delays in referral-to-treatment waiting times for elective admissions of adults from deprived areas and ethnic minority backgrounds: a study of healthcare inequalities. J Racial Ethn Health Disparities. (2025):1–12. doi: 10.1007/s40615-025-02515-5, 40533721 PMC13546329

[7] Mann JR Zhang Y McDermott S Wang Y Cai B Conway KM . Racial and ethnic differences in timing of diagnosis and clinical services received in Duchenne muscular dystrophy. Neuroepidemiology. (2023) 57:90–9. doi: 10.1159/000528962, 36623491 PMC10273877

[8] Sapiets SJ Totsika V Hastings RP. Factors influencing access to early intervention for families of children with developmental disabilities: a narrative review. J Appl Res Intellect Disabil. (2021) 34:695–711. doi: 10.1111/jar.12852, 33354863 PMC8246771

[9] Okoye C Obialo-Ibeawuchi CM Obajeun OA Sarwar S Tawfik C Waleed MS . Early diagnosis of autism spectrum disorder: a review and analysis of the risks and benefits. Cureus. (2023) 15:e43226. doi: 10.7759/cureus.43226 37692637 PMC10491411

[10] Rocha Neto H Moreira ALR Hosken L Langfus JA Cavalcanti MT Youngstrom EA . Inter-rater reliability between structured and non-structured interviews is fair in schizophrenia and bipolar disorders—a systematic review and meta-analysis. Diagnostics. (2023) 13:526. doi: 10.3390/diagnostics13030526, 36766632 PMC9914275

[11] Rahman MM Usman OL Muniyandi RC Sahran S Mohamed S Razak RA. A review of machine learning methods of feature selection and classification for autism Spectrum disorder. Brain Sci. (2020) 10:949. doi: 10.3390/brainsci10120949, 33297436 PMC7762227

[12] Wei Q Xu X Xu X Cheng Q. Early identification of autism spectrum disorder by multi-instrument fusion: a clinically applicable machine learning approach. Psychiatry Res. (2023) 320:115050. doi: 10.1016/j.psychres.2023.115050, 36645989

[13] Rudin C. Stop explaining black box machine learning models for high stakes decisions and use interpretable models instead. Nat Mach Intell. (2019) 1:206–15. doi: 10.1038/s42256-019-0048-x, 35603010 PMC9122117

[14] Topol EJ. High-performance medicine: the convergence of human and artificial intelligence. Nat Med. (2019) 25:44–56. doi: 10.1038/s41591-018-0300-7, 30617339

[15] Jang J. ANFIS: adaptive-network-based fuzzy inference system. IEEE Trans Syst Man Cybern. (1993) 23:665–85. doi: 10.1109/21.256541

[16] Cao J Zhou T Zhi S Lam S Ren G Zhang Y . Fuzzy inference system with interpretable fuzzy rules: advancing explainable artificial intelligence for disease diagnosis—a comprehensive review. Inf Sci. (2024) 662:120212. doi: 10.1016/j.ins.2024.120212

[17] Chen T Shang C Su P Keravnou-Papailiou E Zhao Y Antoniou G . A decision tree-initialised Neuro-fuzzy approach for clinical decision support. Artif Intell Med. (2021) 111:101986. doi: 10.1016/j.artmed.2020.101986, 33461686

[18] Nguyen T Kavuri S Park S Lee M. Attentive hierarchical ANFIS with interpretability for cancer diagnostic. Expert Syst Appl. (2022) 201:117099. doi: 10.1016/j.eswa.2022.117099

[19] Brown KA Parikh S Patel DR. Understanding basic concepts of developmental diagnosis in children. Transl Pediatrics. (2020) 9:S9–S22. doi: 10.21037/tp.2019.11.04, 32206580 PMC7082247

[20] Hyman SL Levy SE Myers SM Kuo DZ Apkon S Davidson LF . Identification, evaluation, and management of children with autism spectrum disorder. Pediatrics. (2020) 145:e20193447. doi: 10.1542/peds.2019-3447 31843864

[21] Estrin GL Milner V Spain D Happé F Colvert E. Barriers to autism Spectrum disorder diagnosis for young women and girls: a systematic review. Rev J Autism Dev Dis. (2020) 8:454–70. doi: 10.1007/s40489-020-00225-8, 34868805 PMC8604819

[22] Hadders-Algra M. Early diagnostics and early intervention in neurodevelopmental disorders—age-dependent challenges and opportunities. J Clin Med. (2021) 10:861. doi: 10.3390/jcm10040861, 33669727 PMC7922888

[23] Rosen NE Lord C Volkmar FR. The diagnosis of autism: from Kanner to DSM-III to DSM-5 and beyond. J Autism Dev Disord. (2021) 51:4253–70. doi: 10.1007/s10803-021-04904-1, 33624215 PMC8531066

[24] Lipkin PH Macias MM Norwood KW Brei TJ Davidson LF Davis BE . Promoting optimal development: identifying infants and young children with developmental disorders through developmental surveillance and screening. Pediatrics. (2019) 145:e20193449. doi: 10.1542/peds.2019-3449, 31843861

[25] Popova S Charness ME Burd L Crawford A Hoyme HE Mukherjee R a S . Fetal alcohol spectrum disorders. Nat Rev Dis Prim. (2023) 9:11. doi: 10.1038/s41572-023-00420-x 36823161

[26] Happé F Frith U. Annual research review: looking back to look forward – changes in the concept of autism and implications for future research. J Child Psychol Psychiatry. (2020) 61:218–32. doi: 10.1111/jcpp.13176, 31994188

[27] Patel DR Neelakantan M Pandher K Merrick J. Cerebral palsy in children: a clinical overview. Transl Pediatrics. (2020) 9:S125–35. doi: 10.21037/tp.2020.01.01, 32206590 PMC7082248

[28] Briguglio M Turriziani L Currò A Gagliano A Di Rosa G Caccamo D . A machine learning approach to the diagnosis of autism Spectrum disorder and multi-systemic developmental disorder based on retrospective data and ADOS-2 score. Brain Sci. (2023) 13:883. doi: 10.3390/brainsci13060883, 37371363 PMC10295931

[29] Song C Jiang Z Hu L Li W Liu X Wang Y . A machine learning-based diagnostic model for children with autism spectrum disorders complicated with intellectual disability. Front Psych. (2022) 13:993077. doi: 10.3389/fpsyt.2022.993077, 36213933 PMC9533131

[30] Haque MM Rabbani M Dipal DD Zarif MII Iqbal A Schwichtenberg A . Informing developmental milestone achievement for children with autism: machine learning approach. JMIR Med Inform. (2021) 9:e29242. doi: 10.2196/29242, 33984830 PMC8262602

[31] Cavus N Lawan AA Ibrahim Z Dahiru A Tahir S Abdulrazak UI . A systematic literature review on the application of machine-learning models in behavioral assessment of autism Spectrum disorder. J Personal Med. (2021) 11:299. doi: 10.3390/jpm11040299, 33919878 PMC8070763

[32] Schulte-Rüther M Kulvicius T Stroth S Wolff N Roessner V Marschik PB . Using machine learning to improve diagnostic assessment of ASD in the light of specific differential and co-occurring diagnoses. J Child Psychol Psychiatry. (2023) 64:16–26. doi: 10.1111/jcpp.13650, 35775235

[33] Rasul RA Saha P Bala D Karim SRU Abdullah MI Saha B. An evaluation of machine learning approaches for early diagnosis of autism spectrum disorder. Healthc Anal. (2024) 5:100293. doi: 10.1016/j.health.2023.100293

[34] Raj S Masood S. Analysis and detection of autism spectrum disorder using machine learning techniques. Procedia Comput Sci. (2020) 167:994–1004. doi: 10.1016/j.procs.2020.03.399

[35] Song C Jiang Z Liu D Wu L. Application and research progress of machine learning in the diagnosis and treatment of neurodevelopmental disorders in children. Front Psych. (2022) 13:960672. doi: 10.3389/fpsyt.2022.960672, 36090350 PMC9449316

[36] Ouifak H Idri A. On the performance and interpretability of Mamdani and Takagi-Sugeno-Kang based neuro-fuzzy systems for medical diagnosis. Sci Afr. (2023) 20:e01610. doi: 10.1016/j.sciaf.2023.e01610

[37] Talpur N Abdulkadir SJ Alhussian H Hasan MH Aziz N Bamhdi A. Deep Neuro-fuzzy system application trends, challenges, and future perspectives: a systematic survey. Artif Intell Rev. (2023) 56:865–913. doi: 10.1007/s10462-022-10188-3, 35431395 PMC9005344

[38] Singh B Doborjeh M Doborjeh Z Budhraja S Tan S Sumich A . Constrained neuro fuzzy inference methodology for explainable personalised modelling with applications on gene expression data. Sci Rep. (2023) 13:456. doi: 10.1038/s41598-022-27132-8, 36624117 PMC9829920

[39] Júnior JSS Mendes J Souza F Premebida C. Survey on deep fuzzy Systems in Regression Applications: a view on interpretability. Int J Fuzzy Syst. (2023) 25:2568–89. doi: 10.1007/s40815-023-01544-8

[40] Nguyen TT Hien DT Nguyen TL. Feature reduction for interpretability of Neuro-fuzzy classifier. In: Nghia PT Thai VD Thuy NT Son LH Huynh VN, editors. Advances in Information and Communication Technology. ICTA 2023. Lecture Notes in Networks and Systems, vol 847. Cham: Springer (2023). p. 186–93. doi: 10.1007/978-3-031-49529-8_20

[41] Chawla NV. Data Mining for Imbalanced Datasets: An Overview. Boston, MA: Springer eBooks (2009). p. 875–86.

[42] Bishop DVM. Which neurodevelopmental disorders get researched and why? PLoS One. (2010) 5:e15112. doi: 10.1371/journal.pone.0015112, 21152085 PMC2994844

[43] Abraham A. Adaptation of fuzzy inference system using neural learning. In: Nedjah N Macedo Mourelle L, editors. Fuzzy Systems Engineering. Studies in Fuzziness and Soft Computing, vol 181. Berlin, Heidelberg: Springer (2005). p. 53–83. doi: 10.1007/11339366_3

[44] Steyerberg EW Vickers AJ Cook NR Gerds T Gonen M Obuchowski N . Assessing the performance of prediction models. Epidemiology. (2010) 21:128–38. doi: 10.1097/ede.0b013e3181c30fb2, 20010215 PMC3575184

[45] Jiang X Osl M Kim J Ohno-Machado L. Calibrating predictive model estimates to support personalized medicine. J Am Med Inform Assoc. (2012) 19:263–74. doi: 10.1136/amiajnl-2011-000291, 21984587 PMC3277613

[46] Holzinger A Langs G Denk H Zatloukal K Müller H. Causability and explainability of artificial intelligence in medicine. Wiley interdisciplinary reviews. Data Min Knowl Disc. (2019) 9:e1312. doi: 10.1002/widm.1312, 32089788 PMC7017860

[47] Centers for Disease Control and Prevention. Data and statistics on children’s mental health. Atlanta, GA: U.S. Department of Health and Human Services, Centers for Disease Control and Prevention. (2022) Available at: https://www.cdc.gov/childrensmentalhealth/data.html (Accessed August 2025).

